# Elevated Circulating Concentrations of Interferon-Gamma in Latent Tuberculosis Infection

**DOI:** 10.20411/pai.v1i2.149

**Published:** 2016-11-01

**Authors:** Moises A. Huaman, George S. Deepe, Carl J. Fichtenbaum

**Affiliations:** 1 Division of Infectious Diseases, Department of Internal Medicine, University of Cincinnati, Cincinnati, Ohio

**Keywords:** latent tuberculosis, interferon gamma, immune activation, NHANES

## Abstract

**Background:**

Latent tuberculosis infection (LTBI) has been associated with increased immune activation. We assessed circulating concentrations of interferon-gamma in persons with LTBI.

**Methods:**

We used the 2011-2012 National Health Nutritional Examination Survey (NHANES) to identify adults with and without LTBI by QuantiFERON®-TB Gold In-Tube (QFT) results. Non-LTBI persons were 1:1 age-, gender-, and race-matched to LTBI persons using propensity scores. We compared the plasma concentrations of interferon-gamma measured from the unstimulated, negative control QFT tube between LTBI and non-LTBI persons. We used Mann-Whitney tests and ordered logistic regressions for comparisons.

**Results:**

There were 430 LTBI and 430 non-LTBI matched persons included in the analysis. LTBI was associated with higher circulating concentrations of interferon-gamma (median, 3 pg/mL; IQR, 2 – 5) compared to non-LTBI (median, 2.5 pg/mL; IQR, 1.5 – 3.5); *P* < 0.001. LTBI remained associated with higher interferon-gamma concentrations after adjusting for age, gender, race, diabetes, hypertension, tobacco use, HIV status, body mass index, lipid profile, and lymphocyte count (odds ratio, 1.79, 95% CI, 1.26 – 2.53). Results remained similar when tuberculin skin testing defined LTBI.

**Conclusions:**

LTBI was associated with increased circulating interferon-gamma concentrations. Future studies are needed to further characterize immune activation in LTBI and its potential long-term consequences.

**STANDFIRST**

Latent tuberculosis infection was associated with increased circulating interferon-gamma concentrations.

## INTRODUCTION

Latent tuberculosis infection (LTBI) affects one-third of the world population [1]. Five to 10% of persons with LTBI develop tuberculosis disease, which carries enormous morbidity and mortality [2]. The other 90% to 95% of persons with LTBI remain asymptomatic, with only those considered at high risk for progression to tuberculosis disease accessing LTBI screening and treatment programs [3, 4].

Increasing evidence shows that, rather than a homogeneous state of dormant infection, LTBI has a wide and dynamic spectrum characterized by host-pathogen interactions that vary over time and tissue microenvironment [5, 6]. There is active replication of *Mycobacterium tuberculosis* restrained by immune responses in chronically infected mice, modeling what likely occurs in human LTBI [7]. Studies in humans suggest that LTBI is associated with increased T-cell activation markers [8, 9]. We assessed circulating concentrations of interferon-gamma (IFN-γ) in persons with LTBI compared to persons without LTBI using a U.S. nationally representative sample of adults with LTBI [10], and taking advantage of the IFN-γ measurements reported from unstimulated, negative control tubes that are part of the QuantiFERON^®^-TB Gold In-Tube (QFT) assay [11].

## METHODS

We used data from the 2011-2012 National Health Nutritional Examination Survey (NHANES) for this project. The NHANES consists of a series of nationally representative, cross-sectional surveys that use a multistage, stratified probability cluster sampling design [12]. The 2011-2012 NHANES survey over-sampled subgroups of Hispanics, non-Hispanic African Americans, non-Hispanic Asians, persons ≤ 130% of the poverty level, and persons aged ≥ 80 years old [13]. For 2011-2012, the survey included tuberculosis questionnaires, tuberculin skin testing (TST) and QFT testing for persons aged ≥ 6 years old. For TST, a positive test was defined as a reading of skin induration ≥ 10 mm 46 to 76 hours after TST administration [10]. A minor variation from the routinely used 48 to 72 hours TST reading window was allowed to facilitate patient scheduling. The QFT assay was performed and interpreted following the Centers for Disease Control and Prevention (CDC) guidelines [14]. Briefly, ~1 mL of blood was drawn into each of three blood collection tubes: a negative control tube (containing heparin alone), a TB antigen tube (containing 3 *M. tuberculosis* antigens: ESAT-6, CFP-10, and TB 7.7), and a positive control tube (containing the T-cell mitogen phytohemagglutinin). The tubes were incubated at 37 °C ± 1°C for 16 to 24 hours. The tubes were transported to a central laboratory at the University of Washington, Seattle where blood specimens were centrifuged and the supernatant plasma isolated. The concentration of IFN-γ in plasma from each of the QFT tubes was determined by enzyme-like immunosorbent assay (ELISA). For interpretation of results, the IFN-γ value in the negative control tube was subtracted from the TB antigen tube value to adjust for background IFN-γ. TB antigen samples with ≥ 0.35 IU/mL IFN- γ were considered positive, while samples with < 0.35 IU/mL were considered negative. The results were considered indeterminate if the concentration of IFN-γ was < 0.35 IU/ mL for TB antigens and < 0.5 IU/mL for the positive control [11].

To examine the relationship between LTBI and circulating IFN-γ levels, we first identified all adult persons ≥ 18 years of age who were diagnosed with LTBI based on a positive QFT test. We then identified those without LTBI based on a negative QFT test to establish a comparison group. Based on information from the tuberculosis questionnaire, we excluded those who reported a prior history of active tuberculosis disease or prior LTBI treatment regardless of whether treatment had been completed or not. Among persons in the comparison group (negative QFT test result), we excluded those who reported a prior history of positive TST, positive blood tuberculosis test, or positive tine test. We used propensity scores for 1:1 matching of LTBI and non-LTBI persons by age, gender, and race using the nearest neighbor method with a caliper width set at 0.2 [15]. We retrieved available information on common medical conditions and metabolic markers that could affect systemic inflammation and immune activation, including history of diabetes mellitus, history of hypertension, and current tobacco use. History of diabetes mellitus was defined as answering yes to any of the following 3 survey questions: 1) were you told that you have diabetes by a doctor? 2) are you currently taking insulin? 3) are you taking blood-sugar lowering pills? History of hypertension was defined as answering yes to any of the following 2 survey questions: 1) were you told at 2 or more different visits that you had hypertension, also called high blood pressure? 2) because of high blood pressure/hypertension, have you ever been told to take prescribed medicine? Current tobacco use was defined as answering yes to the question: do you now smoke cigarettes? We also extracted human immunodeficiency virus (HIV) antibody testing results. Serum specimens were first tested by ELISA. Positive ELISA results were confirmed by Western blot. Additionally, we extracted body mass index (BMI) values obtained during NHANES body examination and laboratory results of fasting glucose in mg/mL, hemoglobin A1c percentage, total cholesterol in mg/dL, triglycerides in mg/dL, direct high-density lipoprotein (HDL) in mg/dL, and direct low-density lipoprotein (LDL) in mg/dL. Because IFN-γ is produced by lymphocytes, we retrieved available absolute lymphocyte counts/uL from hemogram results. Finally, we extracted the values of IFN-γ measured in the negative control QFT tube. We converted the IFN-γ value from IU/mL to pg/mL by dividing the IU/mL value by 0.02 in order to maintain consistency with results presented in the metric system and make our results comparable to most of the literature reporting IFN-γ measurements.

We used the median and interquartile range (IQR) for numeric variables and percentages for categorical variables as measures of central tendency. For unadjusted comparisons between the LTBI and non-LTBI groups, we used the Mann-Whitney-Wilcoxon test and the chi-square test for numeric and categorical variables, respectively. For multivariable analysis we used ordered logistic regressions with IFN-γ as the dependent variable and age, gender, race, hypertension, diabetes mellitus, current tobacco use, HIV status, BMI, hemoglobin A1c, total cholesterol, triglycerides, HDL, LDL, and absolute lymphocyte count as independent variables in the final models, clustered by matched pairs. The results of ordered logistic regressions were reported as adjusted odds ratios accompanied by 95% confidence intervals (95% CI). Non-parametric tests were used for our primary analyses because we expected a non-normal distribution of IFN-γ values. As a supplementary analysis, we calculated means and standard deviations (SD) of log-transformed IFN-γ values for the LTBI and non-LTBI groups and compared them using the T test. Sensitivity analyses using LTBI definitions based on the combination of available TST and QFT results were performed to account for a potential effect of circulating IFN-γ levels on the QFT assay results. All analyses were performed in Stata software (version 12.0; StataCorp, Texas) with *P* < 0.05 as the level of statistical significance. This project was exempt from institutional review board review as it involved the analysis of publicly available, existing, de-identified data.

## RESULTS

There were a total of 4,950 NHANES participants aged 18 years or older with valid QFT results. Of these, 430 (8.7%) had a positive QFT test result and were classified as LTBI; 4,499 (90.9%) had a negative QFT test result and were classified as non-LTBI; and 21 (0.4%) had an indeterminate QFT test result and were therefore excluded from the analysis. The 430 persons with LTBI were 1:1 propensity-score matched to non-LTBI persons using age, gender, and race. The characteristics of the study populations before and after matching are shown in Table 1. Hypertension, diabetes, obesity, tobacco use, and metabolic markers were similarly distributed in the LTBI and non-LTBI groups after matching.

**Table 1.** Characteristics of the study population

Characteristic	Before matching			After matching		
	No LTBI^a^ (n = 4,499)	LTBI^a^ (n = 430)	*P* value^b^	No LTBI^a^ (n = 430)	LTBI^a^ (n = 430)	*P* value^b^
Age in years	45 (30–61)	59 (45–68)	<0.001^c^	58 (45–68)	59 (45–68)	0.966
Male sex	2,206 (49)	254 (59.1)	<0.001^c^	251 (58.4)	254 (59.1)	0.835
Race			<0.001^c^			
Mexican American	432 (9.6)	64 (14.8)		63 (14.7)	64 (14.8)	0.999
Other Hispanic	423 (9.4)	78 (18.1)		81 (18.8)	78 (18.1)	
Non-Hispanic White	1,807 (40.2)	63 (14.7)		63 (14.7)	63 (14.7)	
Non-Hispanic Black	1,183 (26.3)	95 (22.1)		93 (21.6)	95 (22.1)	
Other including multi-race	654 (14.5)	130 (30.2)		130 (30.2)	130 (30.2)	
Hypertension	1,387 (30.8)	155 (36.1)	0.026^c^	178 (41.4)	155 (36.1)	0.107
Diabetes mellitus	539 (11.9)	78 (18.1)	<0.001^c^	75 (17.4)	78 (18.1)	0.789
Current smoker	854 (18.9)	86 (20)	0.608	66 (15.4)	86 (20)	0.074
HIV antibody testing			<0.001^c^			
Positive	14 (0.3)	—		—	—	
Negative	3,141 (69.8)	—		—	—	
Not done	1,344 (29.9)	—		—	—	
Body mass index (BMI), kg/m^2^	27.6 (23.8–32.2)	27.2 (24.1–31.7)	0.641	27.7 (24.3–32)	27.2 (24–31.7)	0.205
Weight categories by BMI			0.665			0.228
Underweight (BMI <18.5)	93 (2.1)	9 (2.1)		3 (7.1)	9 (2.1)	
Normal weight (BMI 18.5–24.9)	1,364 (30.8)	136 (31.9)		118 (27.9)	136 (31.9)	
Overweight (BMI 25–29.9)	1,390 (31.4)	142 (33.3)		152 (35.9)	142 (33.3)	
Obesity (BMI 30–39.9)	1,284 (28.9)	117 (27.5)		121 (28.6)	117 (27.5)	
Morbid obesity (BMI ≥40)	298 (6.7)	22 (5.2)		29 (6.9)	22 (5.2)	
Fasting glucose (mg/mL)	99 (91–108)	102 (95–118.5)	<0.001^c^	103 (94–116)	102 (95–119)	0.857
Hemoglobin A1c, %	5.5 (5.2–5.9)	5.7 (5.4–6.2)	<0.001^c^	5.7 (5.4–6.1)	5.7 (5.4–6.2)	0.710
Hemoglobin A1c ≥6.5%	481 (10.7)	81 (18.8)	<0.001^c^	72 (16.7)	81 (18.8)	0.422
Total cholesterol, mg/dL	188 (162–216)	192 (168–222)	0.019^c^	193 (166–249)	192 (168–222)	0.662
Direct HDL	50 (42–61)	49 (41–59)	0.029^c^	49 (41–57)	49 (41–59)	0.985
Direct LDL, mg/dL	111 (87–135)	111 (89–138)	0.874	113 (92–137)	111 (89–138)	0.341
Triglycerides, mg/dL	100 (72–147)	111 (82–148)	0.034^c^	114.5 (81–157)	111 (82–148)	0.621
Total cholesterol >200 mg/dL	1,768 (39.3)	192 (44.7)	0.030	190 (44.2)	192 (44.7)	0.891
Total LDL >130mg/dL	612 (28.6)	59 (29.8)	0.724	65 (30.5)	59 (29.8)	0.874
Total HDL <40 mg/dL	774 (17.2)	86 (20)	0.144	75 (17.4)	86 (20)	0.336
Triglycerides >150 mg/dL	517 (23.7)	48 (24)	0.933	62 (28.7)	48 (24)	0.277
Lymphocyte count, 1000 cells/uL	2 (1.6–2.4)	2 (1.6–2.4)	0.523	1.9 (1.6–2.4)	2 (1.6–2.4)	0.154

a Data presented as number of observations (%) or median (interquartile range)

b Chi-square test for categorical variables; Mann-Whitney test for numeric variables

c *P* <0.05, statistically significant

**Figure 1.**
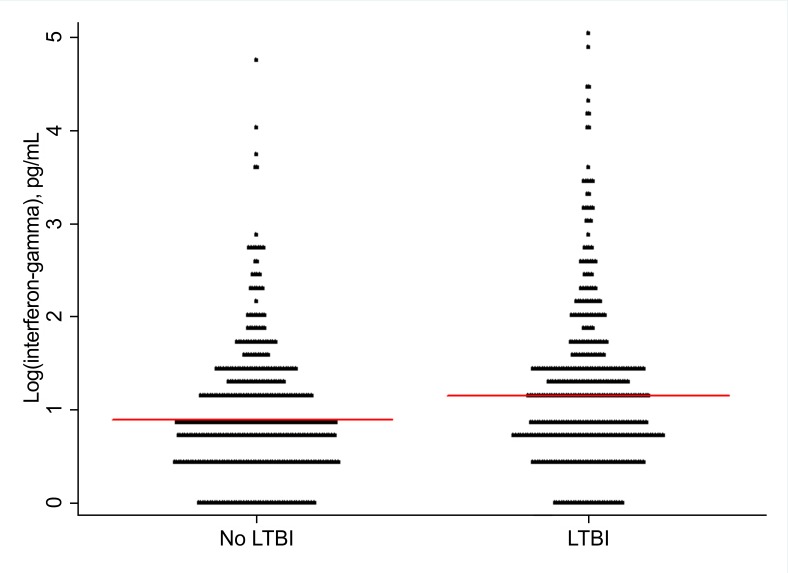
Interferon-gamma concentrations by latent tuberculosis infection (LTBI) status. The median is indicated with the red lines.

LTBI was associated with higher levels of circulating IFN-γ compared to non-LTBI before matching (median [IQR]; 3 pg/mL [2 – 5] vs. 2 pg/mL [1.5 – 3.5]; *P* < 0.001) and after matching (median [IQR]; 3 pg/mL [2 – 5] vs. 2.5 pg/mL [1.5 – 3.5]; *P* < 0.001). Figure 1 shows the distribution of IFN-γ levels in the LTBI and non-LTBI groups after logarithmic transformation (mean ± SD; 1.25 ± 0.87 vs. 0.96 ± 0.72; T-test, *P* < 0.001; median [IQR]; 1.1 [0.69 – 1.61] vs. 0.92 [0.41 – 1.25]; *P* < 0.001) in the matched population.

In multivariable analysis of the matched population, LTBI remained associated with higher IFN-γ concentrations after adjusting for age, gender, race, history of diabetes mellitus, history of hyper-tension, current tobacco use, HIV status, BMI, total cholesterol, triglycerides, HDL, LDL, and lymphocyte count (adjusted odds ratio, 1.79, 95%CI, 1.26 – 2.53; *P* = 0.001). Table 2 shows the complete results of the final ordered logistic regression model for IFN-γ concentrations. Hispanic race, other race, and higher lymphocyte counts were independently associated with higher IFN-γ concentrations. Current tobacco use was independently associated with lower IFN-γ concentrations.

**Table 2.** Results of ordered logistic regression for interferon-gamma concentration as the dependent variable

Variable	Adjusted odds ratio (95% CI)
Latent tuberculosis infection	1.79 (1.26 – 2.53)^a^
Male gender	1.01 (0.69 – 1.47)
Age in years	0.99 (0.98 – 1.02)
Race (reference, non-Hispanic White)	
Non-Hispanic Black	1.14 (0.59 – 2.23)
Mexican American or other Hispanic	2.58 (1.46 – 4.58)^a^
Other	2.4 (1.31 – 4.39)^a^
History of hypertension	0.95 (0.63 – 1.44)
History of diabetes mellitus	0.83 (0.49 – 1.39)
Body mass index, kg/m^2^	0.97 (0.94 – 1.01)
HIV antibody testing result (reference, negative)	
Positive	—
Not done	1.27 (0.71 – 2.29)
Current tobacco smoker	0.59 (0.37 – 0.96)^a^
Lymphocyte count, cells/uL	2.41 (1.74 – 3.36)^a^
Total cholesterol, mg/dL	0.99 (0.34 – 1.84)
Triglycerides, mg/dL	1.01 (0.89 – 1.14)
Direct HDL, mg/dL	1.01 (0.54 – 1.86)
Direct LDL, mg/dL	1.01 (0.54 – 1.86)

a *P* <0.05, statistically significant.

To account for a potential effect of circulating IFN-γ levels on the QFT assay results, we conducted sensitivity analyses using TST results to redefine LTBI in our matched population. Among the 430 adults classified as LTBI by QFT results, 173 (40.2%) had a positive TST, 174 (40.5%) had a negative TST, and 83 (19.3%) had no available TST results. Among the 430 adults classified as non-LTBI by QFT results, 343 (79.8%) had a negative TST, 25 (5.8%) had a positive TST, and 62 (14.4%) had no available TST results. When LTBI was defined as a positive TST and non-LTBI was defined as a negative TST regardless of QFT test results, LTBI by TST remained associated with higher IFN-γ levels in the unadjusted (median [IQR]; 3 pg/mL [2 – 5] vs. 2.5 pg/mL [1.5 – 4]; *P* < 0.001) and adjusted analyses (odds ratio, 1.69, 95% CI, 1.11 – 2.59; *P* = 0.015). When LTBI was defined as having both TST and QFT positive tests, and non-LTBI was defined as having both TST and QFT negative tests, LTBI by concordant TST and QFT results remained associated with higher IFN-γ levels in the unadjusted (median [IQR]; 3 pg/mL [2 – 5] vs. 2.5 pg/mL [1.5 – 3.5]; *P* = 0.001) and adjusted analyses (odds ratio, 2.02, 95% CI, 1.25 – 3.27; *P* < 0.001).

## DISCUSSION

We showed that LTBI is associated with higher levels of IFN-γ concentrations compared to non-LTBI in a large sample of U.S. adults. This association remained after adjusting for several potential confounders, regardless of whether LTBI was defined by TST and/or QFT results.

IFN-γ is primarily produced by activated CD4 and CD8 T cells [16]. It is also produced by natural killer cells and type 1 innate lymphoid cells. IFN-γ has a central role in pathogen-directed immunity and monocyte/macrophage activation [17, 18]. The source of plasma IFN-γ in our study population was unclear. Whether it reflects systemic IFN-γ levels, peripheral blood cell cytokine expression at time of blood draw, or production of IFN-γ during the 16 to 24 hours of tube incubation requires further study. Since LTBI has been associated with markers of CD4 and CD8 T-cell activation in the peripheral blood of persons with and without HIV co-infection [8, 9], it is possible that these cells may be a source of IFN-γ. Elevated serum levels of interleukin (IL)-1β, IL-8, IL-22, and TNF-α have been described in LTBI patients compared to healthy controls [19], and our data add to the repertoire of circulating cytokines found in LTBI patients. Because of the overlap in IFN-γ levels observed between LTBI and non-LTBI patients, our results indicate that levels of this cytokine in the negative control QFT tube cannot be used to discriminate between the two groups in clinical practice.

Overrepresentation of interferon responses has been identified by RNA sequencing from the blood of South African adolescents who are HIV-negative with LTBI and who later develop tuberculosis disease [20]. This signature was independently validated in a cohort of HIV-negative Gambian and South African persons aged 10 to 60 years of age who had household contacts with a positive-smear case of pulmonary tuberculosis. The interferon module included genes involved in type 1 and type 2 interferon responses [21–25]. We found higher circulating levels of type 2 interferon (IFN-γ) in LTBI, but we did not measure type 1 interferons or disease progression by interferon levels. These could be assessed in future prospective studies.

One of the potential drivers of immune activation in LTBI may be caused by low-level *M. tuberculosis* replication. *M. tuberculosis* actively replicates in chronically infected mice, modeling what may be occurring in human LTBI [7]. The intensity of host-pathogen interactions in LTBI appears to be dynamic and may change over time depending on several factors including pathogen burden, virulence factors, host immune system status, and re-exposure events [5]. Variability in the interaction between host and pathogen occurs not only systemically but also in the tissue micro-environment. In non-human primates chronically infected with *M. tuberculosis*, each granuloma has a different number of bacilli and, correspondingly, a varied immune response profile. Collectively, these distinctions indicate that LTBI is actually not a single entity but rather a composite of many different responses in tissues [6].

The absolute lymphocyte count was independently associated with IFN-γ levels. This is expected, because IFN-γ is produced by T lymphocytes. Current tobacco use was associated with lower quantities of this cytokine in the circulation. Tobacco smoking has been associated with increased immune activation, but also with impaired T-cell function, T-cell exhaustion and decreased production of cytokines including IFN-γ [26]. Extracts of cigarette smoke are known to inhibit the production of IFN-γ by peripheral mononuclear cells and smoking enhances Th2 related responses [27, 28]. Increased susceptibility to tuberculosis disease among cigarette smokers may be related in part to reduced T-cell responses and decreased IFN-γ production [29].

Chronic immune activation has been linked to numerous long-term negative effects including cardiovascular disease. In HIV-infected persons, immune activation is considered a major contributor to excess acute myocardial infarction (AMI) and stroke events [30]. No study has systematically assessed the relationship between LTBI and clinical outcomes of chronic immune activation. Interestingly, recent population-based studies have shown increased subsequent risk of stroke and acute coronary syndrome (a composite of AMI and unstable angina) in persons with active tuberculosis [31, 32]. Research is needed to gain insight into the mechanisms driving these associations and to the impact, if any, of LTBI on the progression of cardiovascular disease [33].

Our study had limitations. We used IFN-γ measured in the negative control QFT tube for our analysis of circulating IFN-γ concentrations. Although this approach has not been previously validated, we reason that it may provide an approximation to the actual circulating IFN-γ levels in the patients. The negative control QFT tube contains only heparin. Like the other tubes of the assay, it was incubated at 37 ^°^C ± 1^°^C for up to 24 hours prior to plasma separation and processing [11]. IFN-γ is stable in unprocessed heparinized blood samples stored at room temperature for 24 hours [34], but we cannot be certain that it is stable at 37 °C. It is possible that T cells may be a source of production of IFN-γ in the negative control QFT tube during incubation, thus contributing to the measured IFN-γ levels. Even if the stability of IFN-γ had been affected by the time blood was processed, we have no reason to suspect that this could have been channeled differently in LTBI vs. non-LTBI persons. NHANES did not collect other markers of immune activation, hence we could not correlate the IFN-γ results with circulating levels of other pro-inflammatory cytokines or surface markers of lymphocyte activation. Elevated IFN-γ-mediated immune activation has been described in coronary artery disease, acute coronary syndrome, and enlarged abdominal aortic aneurysms [35–37]; however, to our knowledge, IFN-γ has not been validated as a marker of cardiovascular outcomes. Finally, there is potential misclassification of LTBI in the absence of a gold standard for its diagnosis. We used the TST and QFT tests, which are recommended for establishing LTBI diagnosis per national and international guidelines [3, 4]. In fact, the prevalence of LTBI in the U.S. was recently estimated from 2011-2012 NHANES data [10]. We analyzed IFN-γ levels in LTBI groups defined by QFT and/or TST results and we found similar results.

In conclusion, LTBI was associated with increased circulating IFN-γ concentrations. Future studies are needed to further characterize immune activation in LTBI and its potential long-term consequences.
